# Evaluation of Xpert MTB/RIF assay for detection of *Mycobacterium tuberculosis* in stool samples of adults with pulmonary tuberculosis

**DOI:** 10.1371/journal.pone.0203063

**Published:** 2018-09-13

**Authors:** S. M. Mazidur Rahman, Umme Tasnim Maliha, Shahriar Ahmed, Senjuti Kabir, Razia Khatun, Javeed A. Shah, Sayera Banu

**Affiliations:** 1 Infectious Diseases Division, icddr,b 68, Shaheed Tajuddin Ahmed Sarani, Dhaka, Bangladesh; 2 Department of Medicine, University of Washington, Seattle, Washington, United States of America; 3 VA Puget Sound Health Care System, Seattle, Washington, United States of America; National Institute for Infectious Diseases (L. Spallanzani), ITALY

## Abstract

**Background:**

The Xpert MTB/RIF (Xpert) assay technology allows rapid and sensitive diagnosis of pulmonary tuberculosis (PTB) from sputum specimens. However, diagnosis of PTB is difficult for patients who cannot produce sputum. The objective of this study was to investigate the use of Xpert assay for successful detection of PTB using stool samples from adult subjects.

**Methods:**

Both stool and sputum samples from known smear and Xpert positive PTB patients were collected from a TB hospital in Dhaka. Stool samples were collected from healthy individuals without TB symptoms from a slum area of Dhaka. Stool and sputum samples were decontaminated and concentrated using NALC-NaOH-Na-citrate solution and the resultant sediment was used for Xpert, acid-fast bacilli (AFB) microscopy and culture.

**Results:**

A total of 102 stool samples were collected from PTB patients and another 50 stool samples from healthy individuals without TB. The sensitivity of the Xpert assay for detection of *M*. *tuberculosis* in stool samples of PTB patients was 90.2% (95% CI, 82.9–95.0). All 50 stool samples from healthy individuals were negative by the assay (Specificity 100%; 95% CI, 92.9–100). Compared with the sputum culture positive results the sensitivity of the stool Xpert assay was 94.8% (95% CI, 88.5–97.8). Moreover, stool Xpert demonstrated full concordant results with the sputum culture for detection of rifampicin susceptibility. The cycle threshold values of *rpoB* probes obtained from Xpert assay correlated significantly with the bacilli load in the corresponding stool (Spearman correlation = -0.40, *P* < 0.01) and sputum (Spearman correlation = -0.77, *P* < 0.01) samples as determined by microscopy.

**Conclusions:**

Stool Xpert can be applied as a potential alternative of sputum testing for detection of *M*. *tuberculosis* and accurate determination of RIF susceptibility in adult PTB patients. The assay would be beneficial for rapid diagnosis of PTB for those adult patients who cannot expectorate sputum.

## Introduction

Tuberculosis (TB) is a lethal infectious disease caused by *Mycobacterium tuberculosis*, which is the leading cause of death from a single infectious agent worldwide [1]. According to World Health Organization (WHO), in 2016 there were an estimated 10.4 million new TB cases and 1.3 million TB deaths [1]. In Bangladesh, TB continues to be an important contributor to overall disease burden, killing an estimated 66,000 people per year [1]. Therefore, early detection and determining the anti-TB drug susceptibility is essential for successful treatment and control of the disease. Failure to quickly diagnose and treat the drug resistant TB patients, particularly multidrug resistant (MDR) and extensively drug resistant (XDR) patients leads to increased mortality, development of resistance to additional anti-TB drugs and transmission [2, 3].

In resource poor settings with high TB burden, there is an urgent need for implementing a new rapid diagnostic method for detecting TB. Diagnosis of *M*. *tuberculosis* and determining the drug resistance using solid based culture media is considered as gold standard, but this method is slow and requires about 4–6 weeks to obtain the drug susceptibility results [4]. The Xpert MTB/RIF (Xpert) assay (Cepheid, Sunnyvale, CA) is an automated real-time polymerase chain reaction (PCR) assay, which represents a significant advancement in the detection of TB and rifampicin (RIF) resistance within 2 hours [5, 6]. In 2010, WHO endorsed the implementation of Xpert for National TB Control Programmes (NTP) in developing countries [7]. The Xpert technology allows rapid and relatively sensitive diagnosis of pulmonary TB (PTB) in sputum specimens [8, 9]. However, diagnosis of PTB is difficult for patients who cannot produce sputum, a problem that is particularly common in young children, HIV-positive and elderly patients [10]. Sputum induction, gastric or nasopharyngeal aspiration, or fiber-optic bronchoscopy may all be used to retrieve pulmonary secretions from patients who cannot produce sputum, but these methods are invasive and involve uncomfortable procedures with relatively low yield [11–13]. Thus, a noninvasive method for diagnosing PTB without sputum would improve care for these important patient populations.

PTB patients may swallow sputum, passing mycobacteria from sputum through the gastro-intestinal (GI) tract. Thus, clinicians may detect mycobacterial DNA in stool samples by using molecular techniques. As a noninvasive clinical specimen, stool is considered to be available, easy for collection and has the potential to be used for detection of *M*. *tuberculosis* [14]. Mycobacteria have been detected in stool samples successfully by using microscopy, culture and/or PCR [15–19]. Xpert assay has been utilized for detection of PTB using stool samples from children with various sensitivities ranging from 25–68% [20–24]. Variation of sensitivities might have arisen from the differences of processing of stool samples since effective processing would eliminate the PCR inhibitors and concentrate the number of bacilli for optimum detection by the Xpert assay. In a recent study, Xpert assay was optimized using larger quantity of stool where measures were also taken to eliminate the PCR inhibitors, and the sensitivity of the assay was increased to 84% for detection of PTB in children [25]. However, there were many steps in the sample processing procedure including filtering of the stool sample which may make the assay difficult to perform in routine basis for high burdened laboratories. A simple method is still required for processing of larger quantity of stool which would increase the sensitivity of Xpert assay for detection of PTB.

In the present study we have evaluated the diagnostic performance of Xpert assay for detection of *M*. *tuberculosis* in stool samples obtained from adult PTB patients. The diagnostic yield of the stool Xpert was assessed against the confirmed PTB patients and healthy individuals without TB, and the gold standard sputum culture results from PTB patients.

## Materials and methods

### Study settings and collection of specimens

The study was conducted between the periods of December 2012 to September 2013. Sputum and stool samples from known smear and Xpert positive PTB patients were collected from an ongoing drug resistant TB surveillance study which was approved by the Research Review Committee and the Ethical Review Committee of International Centre for Diarrhoeal Disease Research, Bangladesh (icddr,b). Smear positive PTB patients were defined as patients with at least one sputum specimen positive for acid-fast bacilli (AFB), including any scanty smear result [26]. These PTB patients were selected from 250 Bedded TB Hospital at Shyamoli, Dhaka. As a control, we collected stool samples from healthy individuals without TB symptoms in a slum area of Mirpur, Dhaka. Participants were enrolled in the study only after receiving informed written consent from them. At least 2.0 gm of fresh stool samples were collected from each subject and stored in sterile stool containers. Immediately after collection, the samples were transferred to Mycobacteriology Laboratory of icddr,b and stored at -20°C until processed and tested by Xpert assay.

### Processing of stool specimens

Approximately, 2 gm of stool was taken into a 50 ml centrifuge tube and equal volume of sterile normal saline (0.9% NaCl) was added to the sample and mixed well by vortexing. Then normal saline was added up to 30 ml mark in the centrifuge tube and incubated at room temperature for 30 minutes. After incubation, 10 ml of supernatant was transferred into a new 50 ml centrifuge tube and then decontaminated and concentrated following the Petroff’s NaOH method [27]. Briefly, equal volume of N-acetyl-L-cysteine (NALC)-NaOH-Na-citrate solution (0.5% NALC, 4% NaOH, and 2.94% Na-citrate) was added into the centrifuge tube and incubated for 20 minutes at room temperature. The tube was then filled with sterile phosphate buffer saline (PBS) (pH 6.8) up to 40 ml mark and centrifuged twice at 3000 g for 20 minutes. After centrifugation, the supernatant was discarded and the resultant sediment was saved for microscopy, culture and Xpert.

### Microscopy and culture of stool specimens

For AFB microscopy, one loop full (10 μl) of concentrated processed stool specimen was smeared on a slide and subjected to Ziehl-Neelsen (Z-N) staining. Each stained slide was examined by microscopy using 1000X magnification with oil immersion and reported as negative, scanty, 1+, 2+ and 3+ as described previously [28]. To estimate the burden of bacilli, each sample slide was examined for 100 microscopic fields and then the total number of bacilli was recorded. For culture, the concentrated processed specimen was re-suspended with 3 ml of PBS, and then 2 loop-fulls of this re-suspended specimen was inoculated on two Lowenstein Jensen (L-J) slants, and incubated at 37°C for 8 weeks. The inoculated L-J slants were examined once per week for visible growth of mycobacterial colonies as well as contaminating bacteria. A specimen was considered culture negative when no growth was observed on either of the two L-J slants within 8 weeks of incubation.

### Xpert assay

One ml of re-suspended processed stool specimen was taken into a new 15 ml centrifuge tube. Xpert testing was then performed as per the manufacturer’s instructions using a 2:1 ratio of Xpert reagent to the sample. Before 2 ml of the inactivated reagent-sample mixture was transferred to the Xpert test cartridge, the centrifuge tube was manually agitated twice during a 15 minutes incubation period at room temperature. Cartridges were inserted into the Xpert system and automatically generated results were read.

### Sputum specimens

Xpert was done on unprocessed sputum specimens following the manufacturer’s instruction. An aliquot of sputum specimens were decontaminated and concentrated following Petroff's NaOH method [27]. After centrifugation, the supernatant was decanted and the resultant sediment was re-suspended in 1.0 ml of PBS. AFB microscopy and culture was performed following the same procedures as described above for the stool specimens. Cultured isolates from sputum were subjected to drug susceptibility testing (DST) using standard proportion method in L-J media containing RIF at 40 μg/ml [4]. An isolate was considered as resistant to RIF when the number of colonies on the drug-containing medium was 1% or more compared with the number of colonies developed on the drug-free medium.

## Data analysis

Information and laboratory data for all participants were entered and analyzed by using Statistical Package for Social Sciences (SPSS) version 20. Univariate analyses were performed to examine the association between demographic and clinical variables of confirmed PTB patients and non-TB healthy individuals. *P*<0.05 was considered as evidence of significant difference. The 95% confidence intervals were calculated by using the OPENEPI website (http://www.openepi.com/Proportion/Proportion.htm). Spearman correlation test was used to calculate the correlation between the Ct values of *rpoB* probes obtained from Xpert assay and the bacilli load in the stool/sputum samples as determined by smear microscopy. Student’s t-Test was used to compare the bacilli load and Xpert *rpoB* Ct values between stool and sputum samples. The number of bacilli in stool/sputum samples was counted in 100 microscopic fields. The ‘zero values’ of smear microscopy were redefined as 0.5.

## Results

A total of 102 stool samples from PTB patients (mean age: 33.4 year; range: 20 to 80 years; male: 64) and another 50 stool samples from non-TB healthy individuals (mean age: 34 years; range: 20 to 60 years, male: 19) were collected (Individual data points are provided in the supporting information file, S1 Dataset). The demographic and clinical characteristics of the study subjects are shown in Table 1.

**Table 1:** Demographic and clinical characteristics of pulmonary TB patients and non-TB healthy individuals.

Characteristics	Pulmonary TB patients (n = 102) n (%)	Non-TB healthy individuals (n = 50) n (%)	P value
Age, year, mean (range)	33.4 (20–80)	34.0 (20–60)	0.788
Male	64 (62.7)	19 (38)	0.007
BMI, mean (range)	17.4 (12.3–23.9)	23.0 (17.2–38.9)	<0.001
Previously diagnosed as TB*	7 (6.9)	0 (0)	0.096
History of TB contact	20 (19.6)	4 (8.0)	0.108
Presenting Symptoms			
Weight loss	102 (100)	3 (6.0)	<0.001
Fever	100 (98.0)	0 (0)	<0.001
Cough > 2 weeks	102 (100)	2 (4.0)	<0.001

* Fisher exact test

TB, tuberculosis; BMI, body mass index

### Comparison of stool smear/culture with stool Xpert

**Fig 1:**
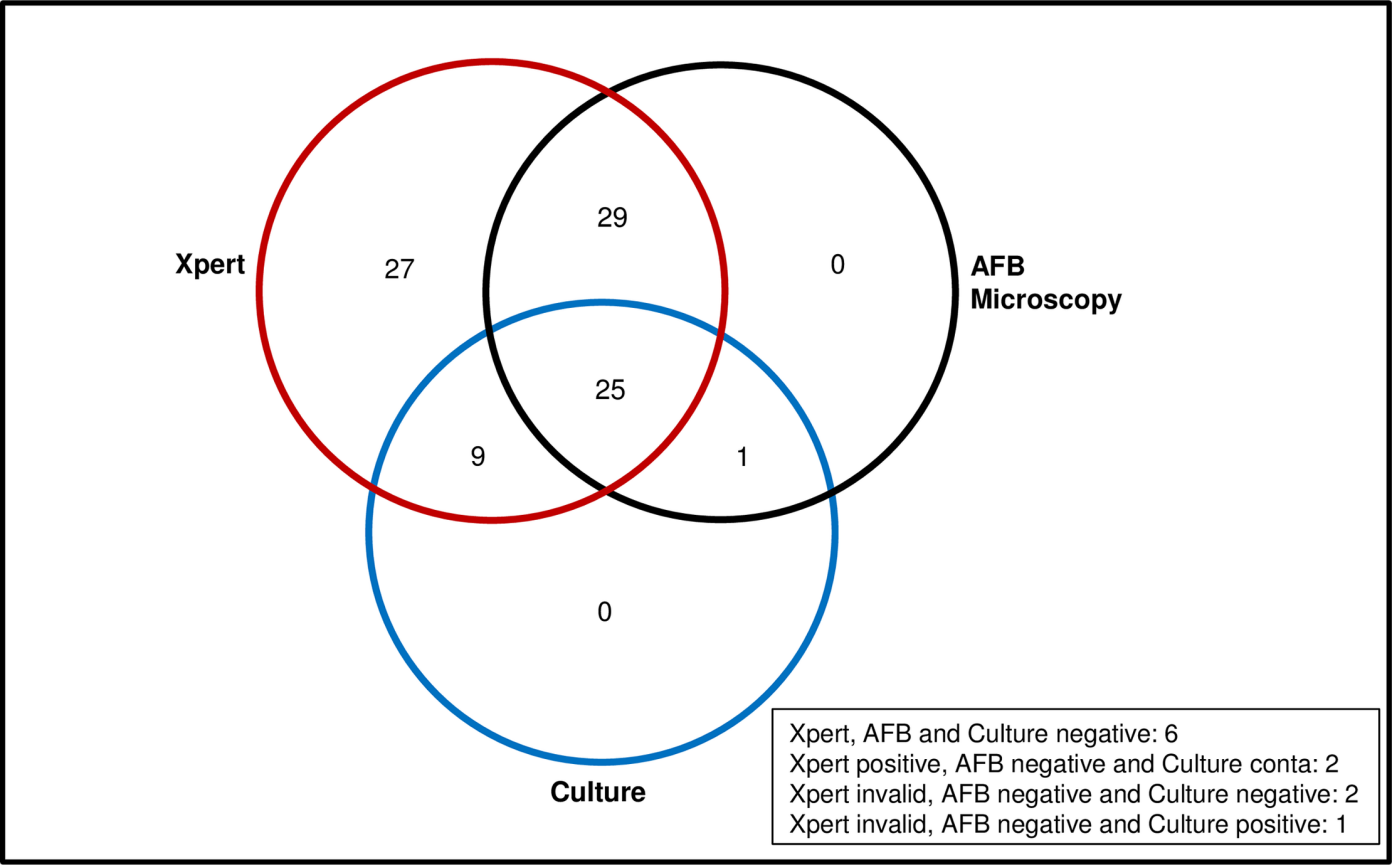
Venn diagram showing the distribution of stool samples for detection of *M*. *tuberculosis* by using Xpert, AFB microscopy and culture. Among 102 stool samples from PTB patients, total 92 were positive for *M*. *tuberculosis* by Xpert assay, whereas, 55 and 36 samples were positive by AFB microscopy and culture, respectively.

Among 152 stool samples tested by Xpert assay, only 3 (2%) samples yielded invalid results for detection of *M*. *tuberculosis*. Delay in the amplification of the internal control (IC) of the Xpert assay is considered to be due to the presence of inhibitors in the specimens [29]. The mean±SD of cycle threshold (Ct) value of the IC of Xpert assay for all stool samples was 30.5±3.8. In total *M*. *tuberculosis* was detected in 92 of the 102 stool samples from patients with PTB (sensitivity, 90.2%; 95% CI, 82.9–95.0). Whereas, all 50 stool samples from non-TB healthy individuals were negative by the Xpert assay (specificity, 100%; 95% CI, 92.9–100). The stool samples were also examined by AFB microscopy and culture in L-J media. Only 2 (1.3%) of 152 stool samples were found contaminated in L-J culture media. The sensitivity of AFB microscopy and culture for detection of *M*. *tuberculosis* in stool samples from PTB patients were 53.9% (95% CI, 44.3–63.3) and 35.3% (95% CI, 26.7–45.0), respectively and specificity for both methods in stool samples from non-TB healthy individuals was 100% (95% CI, 92.9–100) (Table 2). Compared with AFB microscopy and culture, Xpert was more effective in detecting *M*. *tuberculosis* in stool samples. Among 102 stool samples from PTB patients, Xpert detected *M*. *tuberculosis* in total 92 (90.2%) samples either as alone or combinedly with AFB microscopy/culture. Whereas, AFB microscopy detected *M*. *tuberculosis* in 55 (53.9%) stool samples combinedly with Xpert/culture, and culture detected *M*. *tuberculosis* in 36 (35.3%) samples combinedly with Xpert/AFB microscopy. There were 25 (24.5%) and 6 (5.9%) stool samples positive and negative for *M*. *tuberculosis*, respectively by all three methods. There was no sample positive by only AFB microscopy or culture method. Two samples were positive by Xpert but contaminated in culture. Finally, three stool samples were invalid by Xpert assay, all of which were AFB microscopy negative and only one was culture positive (Fig 1).

**Table 2:** The sensitivity and specificity of Xpert MTB/RIF assay, microscopy and culture for detection of *Mycobacterium tuberculosis* in stool samples of pulmonary TB patients and non-TB healthy individuals.

Tests	Test result	Pulmonary TB patients (n = 102)	Non-TB healthy individuals (n = 50)	Sensitivity % (95% CI)	Specificity % (95% CI)
**Xpert MTB/RIF**	Positive	92	0	90.2 (82.9–95.0)	100 (92.9–100)
	Negative	7	50		
	Invalid	3	0		
**Microscopy**	Positive	55	0	53.9 (44.3–63.3)	100 (92.9–100)
	Negative	47	50		
**Culture in L-J media**	Positive	36	0	35.3 (26.7–45.0)	100 (92.9–100)
	Negative	64	50		
	Contamination	2	0		

Of 92 Xpert positive stool samples, valid results for RIF susceptibility were obtained in 89 samples, among which 3 (3.4%) were resistant and 86 (96.6%) were sensitive. Indeterminate results for RIF susceptibility were found in 3 (3.3%) stool samples and the detection load of *M*. *tuberculosis* in these three indeterminate samples were very low by Xpert assay. The mean *rpoB* Ct values of these three indeterminate samples were 34.04, 35.14 and 35.8, respectively.

Among 55 AFB microscopy positive stool samples 54 (98.2%) and 26 (47.3%), and among 47 AFB microscopy negative stool samples 38 (80.9%) and 10 (21.3%) were positive for *M*. *tuberculosis* by Xpert and culture, respectively. The burden of *M*. *tuberculosis* in 92 positive stool samples as determined by Xpert assay was medium, low and very low in 10 (10.9%), 45 (48.9%) and 37 (40.2%) samples, respectively. The detection rate of *M*. *tuberculosis* in stool of PTB patients by Xpert assay increased gradually with higher grading of stool AFB microscopy. The detection rate of *M*. *tuberculosis* in microscopy negative and scanty stool samples was 80.9% (38/47) and 95.8% (23/24), respectively. Whereas, the detection rate was 100% (31/31) among 1+, 2+ and 3+ microscopy positive stool samples (S1 Dataset).

### Comparison of sputum smear/culture with stool Xpert

Among 102 sputum samples of PTB patients, 2 were negative by culture on L-J media. Whereas, 3 stool samples yielded invalid results by Xpert. Therefore, total 97 samples were available for comparison of stool Xpert with sputum culture. When compared with the sputum culture results, the stool Xpert detected *M*. *tuberculosis* in 92 of 97 sputum culture positive specimens (sensitivity 94.8%; 95% CI, 88.5–97.8). When the RIF susceptibility results of stool Xpert were compared with the susceptibility results of sputum culture, stool Xpert detected all 3 RIF resistant and 86 RIF sensitive cases as detected by DST on sputum culture (Table 3).

**Table 3:** The sensitivity and specificity of Stool Xpert for detection of rifampicin susceptibility compared with the results of sputum culture.

		RIF susceptibility by sputum culture		Total
		Resistant	Sensitive	
**RIF susceptibility by stool Xpert**	**Resistant**	03	0	03
	**Sensitive**	0	86	86

**Fig 2:**
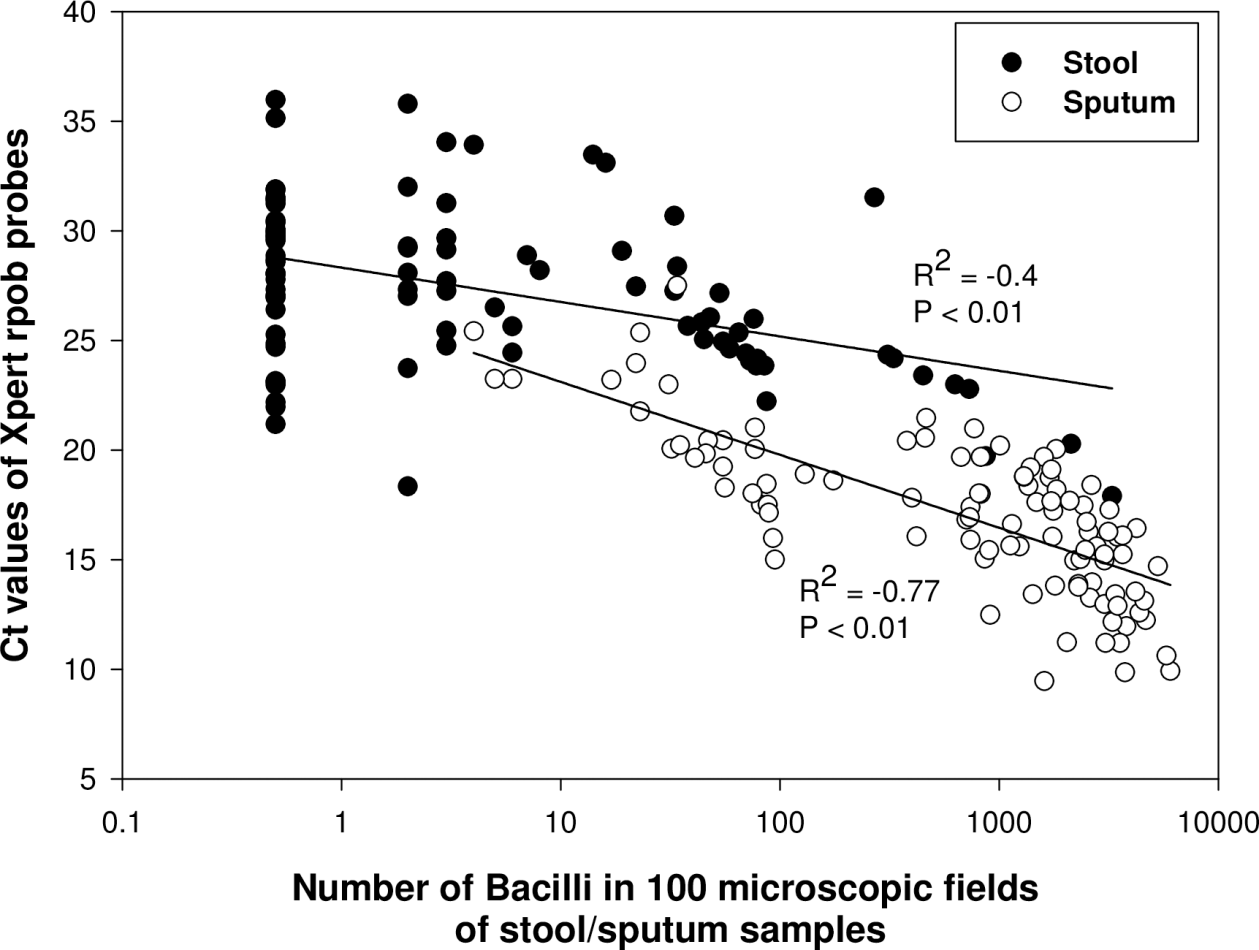
Correlation between the bacilli load and Xpert Ct values in stool/sputum samples. There were significant correlations between the bacillary load as determined by smear microscopy and corresponding cycle-threshold (Ct) values of *rpoB* probes of Xpert MTB/RIF assay for detection of *Mycobacterium tuberculosis* in Xpert positive stool (n = 92)/sputum (n = 102) samples. The bacillary load of each sample was determined by counting the total number of bacilli in 100 microscopic fields. The best-fit lines and spearman regression *R*^*2*^ values are shown.

The cycle threshold (Ct) values of *rpoB* probes obtained from Xpert assay correlated significantly with the bacilli load in the corresponding stool (Spearman correlation = -0.40, *P* < 0.01) and sputum (Spearman correlation = -0.77, *P* < 0.01) samples as determined by AFB microscopy (Fig 2). The Xpert Ct values obtained in sputum samples (mean, 17.1; range, 9.5 to 27.5) were significantly lower compared to the Ct values obtained in stool samples (mean, 27.3; range, 17.9 to 36.0) (*P* < 0.001). Moreover, the bacilli load in 55 microscopy positive stool samples (mean, 185.58; range, 2 to 3280) were significantly lower compared to the bacilli load in corresponding sputum samples (mean, 2108.51; range, 4 to 6050) (*P* < 0.001).

## Discussion

The Xpert assay is used for rapid, sensitive and specific diagnosis of PTB in sputum specimens [8, 9]. However, diagnosis of PTB is difficult for patients who cannot produce sputum [10]. For such cases, diagnosis of PTB using stool specimens has been found to be promising [15–18]. In the present study, we have evaluated the Xpert assay for detection of *M*. *tuberculosis* in stool samples of adults with PTB. The assay demonstrated to be a promising tool as the sensitivity of the assay was 90.2% for detection of *M*. *tuberculosis* in stool samples from confirmed PTB patients. When compared to the gold standard sputum culture results, the sensitivity of the stool Xpert was 94.8%. Sensitivity of the Xpert assay obtained in our study was higher compared to the recently conducted studies which showed a sensitivity of 85.7% using the stool samples from adult PTB cases [30].

The sensitivity of the Xpert assay can vary depending upon the amount of stool used. In previous studies, the sensitivities of the assay varied from 25 to 68% using 0.15 gm of pediatric stool samples [20, 21, 23]. A recent study on analytical spiking experiments of human stool demonstrated that the sensitivity of Xpert increased using 1.0 gm of stool compared to the smaller volume [25]. Further testing with suspected pediatric stool using larger volume, Xpert showed higher sensitivity (84%). In the present study, larger volume of stool (2 gm) was processed for Xpert testing and subsequently higher sensitivity compared to the other studies was achieved [20, 21, 23].

Inhibitors in the fecal constituents often inhibit the PCR amplification due to the inhibition of DNA Taq polymerase [31]. In Xpert testing invalid results are caused due to the delay or absence of Ct of the internal control, which could be due to the presence of PCR inhibitors in the samples [5]. Effective processing of stool samples is essential to eliminate the PCR inhibitors and to obtain valid results. In our study, only 2% of stool samples showed invalid results, and the mean±SD Ct value of IC remained 30.5±3.8 for all stool samples. This suggests that the decontamination of stool samples and washing two times with PBS while processing of sample was effective enough to eliminate the PCR inhibitors. Attempts to eliminate the PCR inhibitors have also been applied in a recent study, where stool samples were treated with commercially available sample processing buffer and then passed through a filter before testing by the Xpert assay. Following this procedure effective elimination of PCR inhibitors was achieved as only 2.6% of stool samples developed invalid results which is similar to our present study [25].

Decontamination and concentration of specimens by NALC-NaOH-Na-citrate solution is considered as a commonly used standard method for detection of mycobacteria [27]. In our previous study we showed that decontamination and concentration of sputum increased the number of mycobacteria and thus increased the sensitivity of microscopy up to 12% compared to the same specimens when performed without processing [28]. By following the same processing method on stool samples we successfully achieved a higher sensitivity. Moreover, Xpert demonstrated a significant correlation with the bacilli load in stool as the Ct values of *rpoB* probes of Xpert decreases with the higher number of bacilli in stool determined by microscopy. Similar correlation was also observed between the bacilli load and Xpert *ropB* Ct values in sputum samples (Fig 2). This finding suggests that the sample processing technique used in this study is highly effective for successful detection of *M*. *tuberculosis* in stool samples of adults by Xpert assay. In another study, based on the principle of floatation technique, salt or sugar solution was used to concentrate *M*. *tuberculosis* in stool samples artificially spiked with known number of bacteria [32]. Although substantial amount of *M*. *tuberculosis* was recovered by Xpert following the floatation technique; the procedure was found more cumbersome and required additional steps. However, processing method used in this study is simpler as it is similar to the procedure widely used in the laboratory for processing of sputum samples.

In addition of detection of *M*. *tuberculosis*, determination of rifampicin susceptibility is crucial for successful treatment of the patient. Our optimized stool Xpert detected all resistant and sensitive cases which were detected by DST on sputum culture. This finding is in agreement with a previous study where stool PCR had high concordance with the culture based testing for determination of rifampicin susceptibility [18]. This entails that in addition of detection of *M*. *tuberculosis*, rifampicin susceptibility can also be determined with high accuracy using stool sample.

In this study, all microscopy positive stool samples were not positive by stool L-J culture. Among 55 microscopy positive stool samples, only 26 (47.3%) were found positive by culture, whereas 54 (98.2%) samples were found positive by Xpert assay. By smear microscopy most of these positive stool samples showed broken bacilli. Passing of swallowed sputum containing tubercular bacilli through highly acidic environment of GI tract might have killed most of the bacteria, but the DNA appeared in the stool. Therefore, not all microscopy positive stool samples yielded positive culture growth, but were positive by stool Xpert. When the bacilli load and Xpert *rpoB* Ct values of sputum and stool samples were compared, stool had significant low load of bacilli and therefore higher Ct values due to lower concentration *M*. *tuberculosis* DNA. This also suggests that the significant numbers of bacilli in sputum are lost during the journey through GI tract, but still can be detected successfully in stool by Xpert. Although less sensitive compared to stool Xpert, stool AFB microscopy and culture could also be applied for detection of *M*. *tuberculosis* from PTB patients who cannot expectorate sputum. This would be particularly helpful for those laboratory settings lacking Xpert but have facility for AFB microscopy and culture.

Diagnosis of PTB in children is always challenging due to the paucibacillary nature of the disease in the lung compared to the adult [33]. Moreover, obtaining good quality respiratory specimens from children can be difficult, but stool can be obtained easily. Xpert on stool samples has been utilized to detect PTB in children with varying degrees of success [21–25]. Currently, we are applying the Xpert assay on stool samples collected from children suspected to have PTB following the optimized sample processing procedure.

The limitation of the study was that the PTB patients were enrolled randomly from an ongoing drug resistance surveillance study, and as a prerequisite of the study all the PTB patients were smear microscopy positive and most of their grading was more than 1+. Therefore, it was not possible to determine whether Xpert could detect sputum smear negative or PTB patients with low bacilli burden using stool samples. All these patients were capable of expectorating sputum; however, the real beneficiary of Stool Xpert would be those who cannot produce sputum but have PTB. Before applying the Xpert assay on stool samples of suspected PTB patients who cannot produce sputum, it is important to know if Xpert can detect *M*. *tuberculosis* from stool sample of bacteriologically positive patients. In this study we aimed to validate the stool processing technique and evaluate the feasibility of detecting *M*. *tuberculosis* from the processed stool specimen of bacteriologically confirmed patients. High performance of stool Xpert in confirmed PTB patients would make the assay more promising for future evaluation on stool samples of suspected PTB patients who cannot produce sputum. Based on our findings, further studies are recommended to unveil the diagnostic performance of this optimized Xpert assay on stool samples of PTB patients who cannot produce sputum. Another limitation of the study was that we had only three RIF resistance cases, and therefore, the sensitivity estimates for detection of RIF resistance had wide range of confidence interval. Finally, we could not exclude the possibility of co-existence of intestinal TB among PTB patients. As the prevalence of co-existent intestinal TB among PTB patients is unknown in Bangladesh, the influence of its presence on the sensitivity of stool Xpert cannot be excluded.

In conclusion, the Xpert assay that we have evaluated performed well for detection of *M*. *tuberculosis* and accurate determination of RIF susceptibility among adult PTB patients using stool samples. The assay would be beneficial for those adults who cannot expectorate sputum, and thereby enhance the rapid diagnosis and treatment of PTB.

## Supporting information

S1 Dataset Individual data points. (XLS)
